# Novel non-helical antimicrobial peptides insert into and fuse lipid model membranes†

**DOI:** 10.1039/d4sm00220b

**Published:** 2024-04-29

**Authors:** Saheli Mitra, Bhairavi Chandersekhar, Yunshu Li, Mark Coopershlyak, Margot E. Mahoney, Brandt Evans, Rachel Koenig, Stephen C. L. Hall, Beate Klösgen, Frank Heinrich, Berthony Deslouches, Stephanie Tristram-Nagle

**Affiliations:** a Biological Physics Group, Physics Department, Carnegie Mellon University Pittsburgh PA 15213 USA stn@cmu.edu; b ISIS Neutron and Muon Source, Rutherford Appleton Laboratory, Harwell Campus Didcot Oxfordshire OX11 0QX UK; c University of Southern Denmark, Dept. Physics, Chemistry & Pharmacy, PhyLife Campusvej 55 Odense M5230 Denmark; d Center for Neutron Research, National Institute of Standards and Technology Gaithersburg MD 20899 USA; e Department of Environmental and Occupational Health, University of Pittsburgh Pittsburgh PA 15261 USA

## Abstract

This research addresses the growing menace of antibiotic resistance by exploring antimicrobial peptides (AMPs) as alternatives to conventional antibiotics. Specifically, we investigate two linear amphipathic AMPs, LE-53 (12-mer) and LE-55 (16-mer), finding that the shorter LE-53 exhibits greater bactericidal activity against both Gram-negative (G(−)) and Gram-positive (G(+)) bacteria. Remarkably, both AMPs are non-toxic to eukaryotic cells. The heightened effectiveness of LE-53 is attributed to its increased hydrophobicity (H) compared to LE-55. Circular dichroism (CD) reveals that LE-53 and LE-55 both adopt β-sheet and random coil structures in lipid model membranes (LMMs) mimicking G(−) and G(+) bacteria, so secondary structure is not the cause of the potency difference. X-ray diffuse scattering (XDS) reveals increased lipid chain order in LE-53, a potential key distinction. Additionally, XDS study uncovers a significant link between LE-53's upper hydrocarbon location in G(−) and G(+) LMMs and its efficacy. Neutron reflectometry (NR) confirms the AMP locations determined using XDS. Solution small angle X-ray scattering (SAXS) demonstrates LE-53's ability to induce vesicle fusion in bacterial LMMs without affecting eukaryotic LMMs, offering a promising strategy to combat antibiotic-resistant strains while preserving human cell integrity, whereas LE-55 has a smaller ability to induce fusion.

## Abstract 2

This research addresses the growing menace of antibiotic resistance by exploring antimicrobial peptides (AMPs) as alternatives to conventional antibiotics.

## Graphical abstract

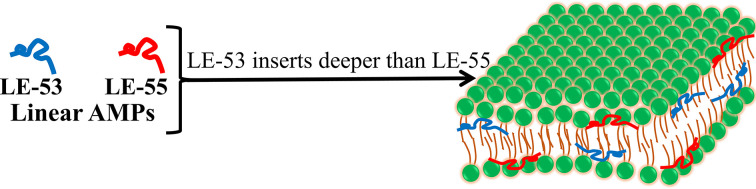

## 1 Introduction

Since their discovery almost a century ago, antibiotics have been hailed as a revolutionary treatment for bacterial infections.^1^ Unfortunately, the effectiveness of antibiotics has been compromised by their excessive use, leading to the development of antimicrobial resistance in a growing number of bacterial strains.^2–4^ This has warranted the exploration of new therapeutic options to combat resistant bacteria, including antimicrobial peptides (AMPs). AMPs, a diverse group of bioactive small proteins, are part of the body's first line of defense for pathogen inactivation. They work by disrupting bacterial cell membranes, modulating the immune response, and regulating inflammation.^5,6^ AMPs being amphipathic (comprising both hydrophilic and hydrophobic parts) readily attach to lipid bilayers characterized by a hydrophilic headgroup and hydrophobic interior. Conventional antibiotics function through diverse mechanisms, such as impeding bacterial cell wall synthesis,^7–9^ DNA replication,^10^ protein synthesis,^11^ or folic acid metabolism.^12^ By contrast, AMPs kill bacteria by perturbing their membrane in a non-specific manner, which delays onset of resistance by several weeks.^13^ Therefore, AMPs could offer an important alternative to traditional antibiotics.^14,15^ It has been shown that the negatively charged phosphate group on lipid A, when modified with phosphoethanolamine (pEtN), renders it neutral. Consequently, the positively charged AMP is no longer attracted to the modified lipid A.^16–18^ This rapid membrane-lytic mechanism grants AMPs a wide range of antimicrobial success, combating susceptible and multi-drug-resistant bacteria. The way AMPs disrupt membranes can vary. This disruption can involve creating pores in the membrane,^19–21^ which can take shapes like “barrel-stave”^22^ or “toroidal”,^15,23^ or it can involve actions like interfacial activity,^24^ thinning the membrane,^25–27^ segregating lipid domains,^28^ or solvation (known as the “carpet” model).^29^ Additionally, Chen *et al.* suggested the “membrane discrimination model,” where the composition of membrane lipids determines how AMPs act; for example, a eukaryotic membrane might cause the same helical AMP to act like a barrel-stave, but a bacterial membrane would facilitate a carpet mechanism.^30^ Bacterial membranes have more negatively charged lipids compared to mammalian cell membranes, so cationic AMPs interact selectively with them.^31,32^

While membrane disruption is often seen as the primary mechanism of action for AMPs, other processes may also contribute to their antibacterial efficacy.^5^ For example, Buforin II, which is structurally similar to the pore forming peptide magainin 2 kills bacteria without cell lysis and has a strong affinity for DNA and RNA, suggesting that Buforin II's target is intracellular nucleic acids, not cell membranes.^33–35^ Other processes such as inhibition of nucleic-acid synthesis,^36^ protein synthesis^37–39^ and enzymatic activity^40,41^ also contribute to antimicrobial peptide effectiveness. Further, several homopolypeptides, such as poly-l-lysine and poly-l-arginine, that belong to the class of cell-penetrating peptides (CPPs), have antibacterial properties.^42–47^ CPPs penetrate the cell membrane through two independent mechanisms: one involving endocytosis and the other membrane translocation,^48–52^ often leaving the membrane intact. However, the precise mechanism of the antibacterial action of CPPs remain incompletely understood.^53^

Recent advancements have demonstrated that we can modify cationic AMPs to improve their effectiveness and selectivity. One way is by carefully selecting specific combinations of amino acids. For instance, incorporating positively charged arginine (Arg, R) residues on one side of the helix and hydrophobic valine (Val, V) residues on the other side can enhance selectivity.^54^ Additionally, extending the peptide chain length and introducing tryptophan (Trp, W) on the hydrophobic side of the peptide can also boost antimicrobial activity.^55–57^ Tryptophan-rich natural AMPs like tritrpticin and indolicidin have shown enhanced potency against a wide range of bacteria while also being less sensitive to salt and serum.^55^ For example, the engineered peptide WLBU2, which is rich in W, exhibits broader antimicrobial activity compared to other available AMPs such as LL-37, polymyxin B, and colistin.^58^ However, a key challenge in designing AMPs is to enhance their antibacterial effects without increasing the risk of harming the host. To address this, we employ a systematic approach to peptide design, adjusting the length and sequence of specific amino acids. Some studies have found that using W exclusively in the hydrophobic domain can enhance both antimicrobial activity and host toxicity due to its high hydrophobicity and bulky indole ring.^55,59^ Thus, in order to mitigate toxicity to eukaryotic cells we added three, four or five Ws to the remaining Vs in the hydrophobic domain.^60,61^

Various scientific methods have been used to study AMPs.^62–65^ For instance, we’ve employed X-ray diffuse scattering (XDS) to observe how colistin affects the elasticity and lipid organization of membranes, hinting at a mechanism involving lipid domains.^66^ Our research has shown that both WLBU2 and its stereoisomer D8-WLBU2 cause similar changes in membrane elasticity as colistin.^67^ One notable aspect of AMPs is their ability to adopt different secondary structures (like α-helix, β-sheet, β-turn, or random coil) when interacting with bacterial membranes, which is crucial for their effectiveness.^68^ We’ve worked to optimize the secondary structure of WLBU2 to form an amphipathic α-helix, enhancing its ability to fight bacteria.^69^ In our recent study involving peptides rich in R and W, such as E2-35 (16 AAs) and E2-05 (22 AAs), we found that the percentage of α-helicity varies depending on the lipid composition of the membranes: greater in G(−) bacteria inner membranes compared to G(+) bacteria or eukaryotic membranes with 33% cholesterol. Our XDS data revealed that a headgroup location correlates with efficacy, but also with toxicity. The membrane bending modulus *K*_C_ displayed non-monotonic changes due to increasing concentrations of E2-35 and E2-05 in G(−) and G(+) LMMs, suggesting a bacterial killing mechanism where lipid domain formation causes ion and water leakage.^70^

The present study aims to compare the antibacterial activity, cytotoxicity and biophysics of two linear amphipathic peptides, LE-53 (12-mer) and LE-55 (16-mer), which are both rich in R and W. We classify them as linear amphipathic due to the linear separation between hydrophilic and hydrophobic residues in their primary sequences (as shown in Table 1). By maintaining this linear separation, formation of a stable helix is prevented. In a helical wheel scheme, hydrophobic and hydrophilic amino acids are intermixed which disrupts the helical structure. The peptide LE-55 has the same amino acid composition as the helical peptide E2-35,^70^ but is designed to not be helical. The secondary structures of the AMPs were analyzed using circular dichroism (CD) measurements to explore potential correlations with their activity. To investigate the structure of the membranes, XDS was employed to explore the location of AMPs within different lipid model membranes, as well as membrane rigidity and lipid chain order. Neutron reflectometry (NR) experiments served to validate the X-ray findings. Additionally, solution small angle X-ray scattering (SAXS) was utilized to study the fusogenic properties of these peptides. Through *in vitro* microbiological assays, the antibacterial activity and cytotoxicity of LE-53 and LE-55 were determined.

**Table 1:** Amino acid sequences of the peptides and their physical attributes. The charged residues are bolded

Peptide	Peptide primary sequence	#AA	Charge	H
LE-53	**RR RR RR** WW WW VV	12	+6	0.448
LE-55	**RR RR RR RR** WW WW VV VV	16	+8	0.362

## 2 Experimental

### 2.1 Materials

The synthetic lyophilized lipids 1-palmitoyl-2-oleoyl-*sn*-glycero-3-phosphoethanolamine (POPE), 1-palmitoyl-2-oleoyl-*sn*-glycero-3-phospho-(10-*rac*-glycerol) sodium salt (POPG), 10,30-bis[1,2-dioleoyl-*sn*-glycero-3-phospho]-*sn*-glycerol sodium salt (TOCL, *i.e.*, cardiolipin), 1-palmitoyl-2-oleoyl-*sn*-glycero-3-phosphocholine (POPC), egg sphingomyelin (ESM), and 1,2-dioleoyl-3-trimeathylammoniumpropane chloride salt (DOTAP) were purchased from Avanti Polar Lipids (Alabaster, AL) and used as received. Cholesterol was from Nu-Chek-Prep (Waterville, MN). HPLC-grade organic solvents were purchased from Sigma-Aldrich (St. Louis, MO). Lipid stock solutions in chloroform were combined to create lipid mixtures in molar ratios mimicking the G(−) Inner Membrane (IM): POPE/POPG/TOCL (7 : 2 : 1 molar ratio), G(+) membrane: POPG/DOTAP/POPE/TOCL (6 : 1.5 : 1.5 : 1),^71^ and eukaryotic membrane, Euk33: POPC/ESM/POPE/cholesterol (15 : 4:1 : 10) (33 mole % cholesterol).^72^ Bacterial cation-adjusted Mueller Hinton Broth (MHB2), Test Condition Media, Roswell Park Memorial Institute (RPMI) media, fetal bovine serum (FBS) and phosphate-buffered saline (PBS) were obtained from Millipore Sigma (St Louis, MO). RPMI media contains the reducing agent glutathione as well as biotin, vitamin B12, and *para* aminobenzoic acid. In addition, RPMI media includes high concentrations of the vitamins inositol and choline. Because RPMI contains no proteins, lipids, or growth factors, it is commonly supplemented with FBS. FBS contains more than 1000 components such as growth factors, hormones, and transport proteins that contribute to cell growth when supplemented into culture media.^73^ Formaldehyde was obtained from Thermo Fisher (Waltham, MA). The peptides LE-53 (*M*_W_: 2610 gm mol^−1^) and LE-55 (*M*_W_: 3313 gm mol^−1^) were purchased in lyophilized form (10 mg in a 1.5 mL vial) from Genscript (Piscataway, NJ) with HPLC/MS spectra corresponding to each designed primary sequence. The traditional antibiotics and colistin were purchased from Millipore Sigma (St. Louis, MO). Peptides’ compositions and their physical attributes are provided in Table 1.

### 2.2 Methods

#### 2.2.1 Antibacterial assay

Bacterial clinical isolates used for initial screening were anonymously provided by the clinical microbiology laboratory of the University of Pittsburgh Medical Center (UPMC). Bacteria were stored at −80 °C and typically retrieved by obtaining single colonies on agar plates prior to subsequent liquid broth culture. Suspensions of test bacteria were prepared from the log phase of growth by diluting overnight cultures at 1 : 100 with fresh cation-adjusted MHB2 and incubating for an additional 3–4 h. Bacteria were spun at 3000*g* for 10 min. The pellet was resuspended in Test Condition Media to determine bacterial turbidity using a Den-1B densitometer (Grant Instruments, Beaver Falls, PA) at 0.5 McFarland units corresponding to 10^8^ CFU mL^−1^.

To examine antibacterial activity, we used minor modifications of a standard growth inhibition assay endorsed by the Clinical and Laboratory Standards Institute (CLSI), as previously described.^74^ Bacteria were incubated with each of the indicated peptides in MHB2. The bacterial cells were kept in an incubator for 18 h at 37 °C, which is linked to a robotic system that feeds a plate reader every hour with one of 8 × 96-well plates. The 96-well plates are standard flat-bottom microliter plates purchased from Thermo Fisher (Waltham, MA). This setup allows the collection of growth kinetic data at A570 (absorbance at 570 nm) to examine growth inhibition in real-time (BioTek Instruments, Winooski, VT). We define minimum inhibitory concentration (MIC) as the minimum peptide concentration that completely prevents bacterial growth, demonstrated by a flat (horizontal line) growth curve as a function of hourly determinations for 18 h. at A570 (Fig. S5, ESI†). The assays are typically repeated a second time. If the MIC differs from the first assay, a third experimental trial is performed to confirm the MIC.

#### 2.2.2 Determination of toxicity to mammalian cells

Toxicity to eukaryotic cells was examined using human red blood cells (RBCs) and peripheral mononuclear cells (PBMC or white blood cells (WBCs)) as previously described.^61,75^ Briefly, RBCs and WBCs were separated by histopaque differential centrifugation using blood anonymously obtained from the Central Blood Bank (Pittsburgh, PA). For the RBC lysis assay, the isolated RBCs were resuspended in PBS at a concentration of 5%. The peptides were serially diluted twofold in 100 μL of PBS before adding 100 μL of 5% RBC to a final dilution of 2.5% RBC to ensure that the A570 of hemoglobin did not saturate the plate reader. In parallel, the RBCs were osmotically burst with water at increasing concentrations to generate a standard curve of RBC lysis. Three technicians independently conducted experiments to ensure reproducibility.

Human WBCs RPMI and 10% FBS were incubated with each selected peptide for 1 h at 37 °C. The cells were then immediately washed with PBS at 1000 *g* for 7 min, while in a round-bottom 96-well plate. After resuspension in PBS, fixable blue live/dead stain from Life Technologies was added according to manufacturer's instructions. The cells were again washed and resuspended in PBS to remove non-specific stain and then fixed with 4% formaldehyde for 1 h. After washing again with PBS, the samples were stored at 4 °C overnight (in the dark) before examination by flow cytometry using the Novocyte flow cytometer (Agilent Technologies, Santa Clara, CA). Peptide-treated cells were compared with untreated cells for dye incorporation, and data were analyzed using the Novocyte analytical software. Dye incorporation was quantified as percent toxicity directly determined by distinguishing live from dead populations,^75^ which was plotted using GraphPad (Prizm software, San Diego, CA).

#### 2.2.3 Circular dichroism (CD)

Unilamellar vesicles (ULVs) of ∼600 Å diameter were prepared using an extruder (Avanti Polar Lipids, Alabaster, AL). 250 μL of 20 mg mL^−1^ lipid in 15 mM PBS was extruded 21 times through a single Nucleopore filter of size 500 Å using 0.2 mL Hamilton syringes. The final concentration of lipid in the ULVs was 18 mg mL^−1^ as determined gravimetrically. Concentrated ULVs were added to 3 mL of 10 μmol L^−1^ (μM) peptide in 15 mM PBS at pH 7 to create lipid/peptide molar ratios between 0 : 1 and 70 : 1. Higher molar ratios of lipid:peptide were not possible due to absorption flattening in the UV region. The samples remained at room temperature for ∼16 hours before the CD measurement. Data were collected in 3 mL quartz cuvettes using a Jasco 1500 CD spectrometer at 37 °C in the Center for Nucleic Acids Science and Technology (CNAST) at Carnegie Mellon University. The samples were scanned from 200 to 240 nm 20 times and the results were averaged. The temperature was controlled at 37 °C *via* a Peltier element and water circulation through the sample compartment. Nitrogen gas was used at a flow rate between 0.56 and 0.71 m^3^ h^−1^ to protect the UV bulb. OriginPro 2019 (OriginLab, Northampton, MA) was used to carry out a Levenberg–Marquardt least squares fit of the tryptophan-subtracted ellipticity traces to four secondary structural motifs representing α-helix, β-sheet, β-turn and random coil.^25,76^ This analysis gives a percentage match of each secondary structural motif to the total sample ellipticity. Instrument ellipticity (*ε*) was converted to Mean Residue Ellipticity using MRE (deg cm^2^ dmol^−1^) = *ε* × 10^4^/*N*, where *N* = # amino acids and peptide concentration was always 10 μM.

#### 2.2.4 Low-angle X-ray diffuse scattering (XDS)

Oriented samples consisting of stacks of approximately ∼1800 bilayers were prepared using the well-established “rock and roll” method.^77^ 4 mg of lipids and peptides in organic solvent, chloroform:methanol (2 : 1, v/v) or trifluoroethanol : chloroform (1 : 1, v/v), were deposited onto a Si wafer (15 mm *W* × 30 mm *L* × 1 mm *H*) inside a fume hood. After rapid evaporation while rocking the substrate, an immobile film formed which was then further dried inside the fume hood for two hours, followed by overnight drying under vacuum to evaporate residual organic solvent. The samples were trimmed to occupy 5 mm *W* × 30 mm *L* along the center of the Si substrate. The substrate was fixed to a glass block (5 mm *H* × 10 mm *W* × 32 mm *L*) using heat sink compound (Dow Corning, Freeland, MI). The sample was stored in a refrigerator at 4 °C for several hours. Cold storage prior to transfer into a well-insulated hydration chamber held at 37 °C caused 100% hydration through the vapor within just 10 minutes. This process is faster than our previous method that required a Peltier cooler under the sample.^78^ Low-angle XDS (LAXS) data from oriented, fully hydrated samples were obtained at the ID7A1 line at Center for High Energy X-ray Sciences (CHEXS, Ithaca, NY) on two separate trips to the Cornell High Energy Synchrotron Source (CHESS) using X-ray wavelengths of 0.8434 Å and 0.8819 Å and sample-to-detector (*S*)-distances of 410 mm and 390.5 mm, with an Eiger 4M detector. Measurements were carried out in the fluid phase at 37 °C. The flat silicon wafer was rotated from −1 to 6 degrees during the data collection at CHESS to vary the angle of incidence. The background was collected by setting the X-ray angle of incidence to −1.7 degree, where sample scattering does not contribute to the image. For data analysis, backgrounds were first subtracted to remove extraneous air and mylar scattering and the images were laterally symmetrized to increase the signal-to-noise ratio. As the sample nears full hydration, membrane fluctuations occur which produce ‘‘lobes’’ of diffuse X-ray scattering data.^79^ The fluctuations are quantitated by measuring the fall-off in lobe intensity in the lateral *q*_r_ direction. The fitting procedure is a non-linear least squares fit that uses the free energy functional from liquid crystal theory,^80^1
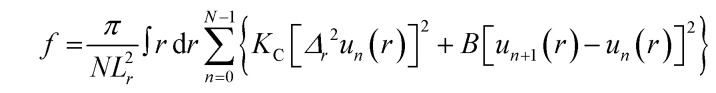
where *N* is the number of bilayers in the vertical (*Z*) direction, *L*_*r*_ is the domain size in the horizontal (*r*) direction, and *K*_C_ is the bending modulus. *K*_C_ describes the bending of an average, single bilayer, *u*_*n*_ is the vertical membrane displacement and *B* is the compressibility modulus. A higher *K*_C_ indicates a stiffer membrane, and a lower *K*_C_ indicates a softer membrane.

#### 2.2.5 Wide-angle XDS

Wide-angle XDS (WAXS) was obtained at CHESS. Before LAXS data is taken, WAXS data is collected as the thin film sample on the Si wafer hydrates. It is X-rayed with a fixed glancing angle of incidence, instead of a rotation of the sample. Two exposures are taken at angles of X-ray incidence *α* = +0.3^0^ and *α* = −0.3^0^, where the negative angle image is then subtracted from the positive angle image. Both are 30-second scans. The subtraction procedure removes extraneous scatter due to the mylar chamber windows and shadows. The chain–chain correlation appears as strong diffuse scatter projecting upwards circularly from the equator; the fall-off in intensity yields information about chain order. To obtain an *S*_xray_ order parameter, the intensity is first integrated along its trajectory then fit to wide-angle liquid crystal theory.^81^ The chain scattering model assumes long thin rods that are locally well aligned along the local director (*n*_L_) with orientation described by the angle *β*. While acyl chains from lipids in the fluid phase are not long cylinders, the model allows the cylinders to tilt (*β*) to approximate chain disorder. From the fit of the intensity data using a Matlab^82^ computer program, we obtain *S*_xray_ using eqn (2):2
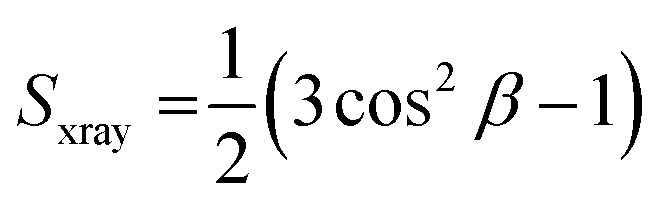


We also obtain the RMSE (root mean square error), which reports the goodness of the fit. An example of LAXS (Fig. S2a, ESI†) and WAXS (Fig. S2b, ESI†) for LE-53 in G(−)IM LMMs at 250 : 1 molar ratio is added in ESI.†

#### 2.2.6 Solution small angle X-ray scattering (SAXS) measurements on ULVs

SAXS measurements were performed on ULVs (prepared as described in Section 2.4) of lipids with embedded peptides using a Xeuss 3.0 (XENOCS, Holyoke, MA) instrument. The instrument features a CuKα rotating anode source (*λ* ∼ 1.5418 Å) and an Eiger 1M detector (Dectris, Switzerland). The system was in the high-flux configuration with a scattering vector (*q*) range of 0.03 < *q* < 0.73 Å^−1^ with sample-to-detector distance = 370 mm. ULVs were robotically injected into the Xeuss BioCube flow cell to enable precise measurements of very small volumes (15 μL). Measurements were carried out at 37 °C with 600 s exposures. Scattering intensity (*I*) *versus* scattering vector *q* (*q* = 4π/*λ* sin(*θ*), where *λ* is the wavelength and 2*θ* is the scattering angle) was obtained by azimuthally averaging the 2D data. As demonstrated in ref. 83, the absorption coefficient by ULV solution is independent of *q* over the range studied; hence, no absorption correction was required. Further, a linear intensity corresponding to pure water was subtracted from the acquired scattering intensity *I*(*q*).

#### 2.2.7 Neutron reflectometry (NR)

NR measurements were performed at the OFFSPEC reflectometer at the ISIS Neutron and Muon Source, Rutherford Appleton Laboratory, Didcot, United Kingdom.^84^ Reflectivity curves were recorded at 37 °C temperature for momentum transfer values 0.01 Å^−1^ ≤ *q*_*z*_ ≤ 0.33 Å^−1^. The neutron sample cells allow *in situ* buffer exchange, and a series of measurements on the same bilayer under different isotopic buffers (pure H_2_O and D_2_O) were performed on the same sample area. 6 mg lipid/peptide mixtures were co-solubilized in chloroform, dried under vacuum and hydrated for 1–2 hours *via* bath sonication in 1.2 mL 2 M NaCl, thereby creating peptide-containing lipid vesicles. Sparsely-tethered lipid bilayer membranes (stBLMs) were prepared on smooth gold-coated (∼140 Å film thickness, 4–9 Å r.m.s surface roughness) silicon wafers that were immersed in a 70 : 30 mol : mol β-mercaptoethanol : HC18 tether solution in ethanol for at least 60 min, leading to the formation of a self-assembled monolayer (SAM) of both molecules at the gold surface.^85^ SAM-decorated wafers were assembled in the NR cell, and lipid bilayers were completed by fusing vesicles of the desired lipid/peptide mixtures using an osmotic shock procedure.^86^ NR data were sequentially collected after rinsing the NR cell with ∼6 cell volumes of either D_2_O or H_2_O using a syringe. NR datasets collected on stBLMs immersed in isotopically different buffers were analyzed simultaneously (2 datasets per stBLM). One-dimensional structural profiles of the substrate and the lipid bilayer along the interface normal *z* were parameterized with a model that utilizes continuous volume occupancy distributions of the molecular components.^87^ Free-form peptide profiles were modeled using Hermite splines with control points on average 15 Å apart.^88^ The protein extension along the membrane normal determines the number of spline control points and was iteratively refined. A Monte Carlo Markov Chain-based global optimizer was used to determine best-fit parameters and their confidence limits.

## 3 Results and discussion

### 3.1 Toxicity to bacteria

LE-53 and LE-55 were initially screened for antibacterial potency against an MDR panel of Gram negative (G(−)) and Gram positive (G(+)) bacterial isolates from UPMC. The MIC is measured by a horizontal growth curve taken every hour;^61^ these MIC values are listed in Table 2. The MICs represent the average of different strains of each type of bacteria. The G(−) bacterial strains are: *Pseudomonas aerginosa (PA)* (*PA14, PA01, PA828*), *Acinetobacter baumannii (AB)* (*ABF8, ABF9, AB-A3, AB-A2*), *Klebsiella pneumoniae (KP)* (*KPC3, KPC3, KP43816, KPB3, KPA5*), *Escherichia coli (EC) (YDC748, YDC337, YDC107, 25922)* and *Enterobacter (Entbac)* (*EA518, EA596, EC470, EC 560)*. The G(+) bacterial strains are: *Enterococci (Entcoc)* (*EF23614, EF24670, EF26215, EF26692*) and *Staphylococcus aureus (SA)* (*SA49775, MRSA-US300*). Notably, the short peptide LE-53 displays broad activity against both G(−) and G(+) bacterial species, outperforming LE-55 and tobramycin (a conventional antibiotic) with the lowest MIC values. The results presented in Table 2 clearly demonstrate a significant variation in MIC values across different bacterial species.

**Table 2:** Antibacterial activity and toxicity of LE-53 and LE-55 peptides

	MIC (μM)									% Toxicity	
	G(−)						G(+)				
	*PA*	*AB*	*KP*	*EC*	*Entbac*	Average	*Entcoc*	*SA*	Average	RBC	WBC
LE-53	10.7	3.3	3.6	4.8	6.5	5.8 ± 1.3	14.4	2.00	8.2 ± 3.0	0	0
LE-55	32.0	21.3	32.0	28.8	32.0	29.2 ± 2.1	28.0	24.0	26.0 ± 1.3	0	0
Colistin	8.4	0.5	0.7	4.3	12.1	5.2 ± 2.2	32.0	64.0	48.0 ± 23	—	—
Tobramycin	32.0	32.0	2.1	28.0	24.5	23.7 ± 3.4	25.0	13.1	19.0 ± 10	—	—

The antimicrobial peptides were examined for minimum inhibitory concentrations (MIC) against G(−) and G(+) MDR isolates from UPMC. % RBC lysis at 32 μM and % toxicity at 16 μM against human WBCs are shown. The MICs are the average of different strains of each type of bacteria. Data are representative of 2–3 experimental trials. See text for bacteria names.

**Fig. 1:**
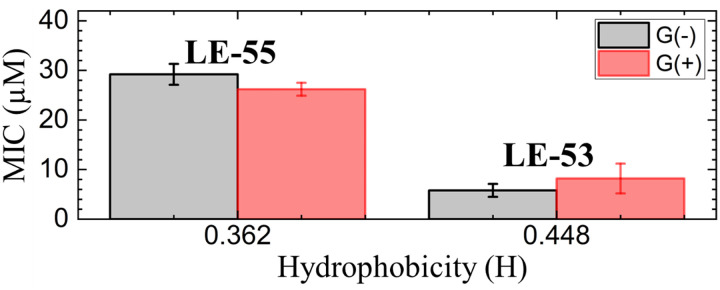
MIC *vs.* hydrophobicity (H) of LE-peptides in G(−) and G(+) bacteria. Errors bars correspond to standard error of the mean values, *σ* = Std. dev/√*N*. Std. dev are calculated by combining the standard deviations for each bacterial species, *σ*_Ave_ = √((*σ*_A_)^2^ + (*σ*_B_)^2^ + (*σ*_C_)^2^…)/*N*.

In order to compare MIC values to physical attributes, we used the HeliQuest website (https://heliquest.ipmc.cnrs.fr)^89,90^ to calculate hydrophobicity (H). The two peptides LE-53 and LE-55 differ in length, charge, and H as detailed in Table 1. Two fewer Rs and two fewer Vs in LE-53 compared to LE-55 increased its H considerably. Fig. 1 compares bactericidal efficiency *vs.* H. Strikingly, a clear correlation between H and bactericidal efficiency was observed. LE-53, characterized by higher values of H, demonstrated significantly lower MICs than LE-55, indicating hydrophobicity could be the cause of higher efficacy. A similar trend was observed by Rosenfeld *et al.* with a group of 12-mer peptides composed of d,l-amino acids and their fatty acid conjugates.^91^ Further, linear amphipathic LE-55 has a similar hydrophobicity with our previously published amphipathic α-helical peptide, E2-35.^70^ However, LE-55 demonstrated inferior antibacterial activity compared to E2-35. Consequently, we believe that altering the primary sequence does indeed impact bactericidal efficiency, while hydrophobicity may play a secondary role.

It is important to note that this trend cannot be universally generalized. Many studies have reported conflicting or inconclusive trends regarding the relationship between H and bactericidal efficiency. In a study by Chen *et al.* MIC displayed a “sweet spot” of hydrophobicity *vs.* efficacy.^30^ In that study, peptide aggregation decreased antibacterial activity as hydrophobicity increased beyond an ideal value.^30^ In another study, antibacterial activity increased above a threshold value of hydrophobicity.^92^ Recently Mitra *et al.* reported poorer efficacy with increasing hydrophobicity for R and W rich helical peptides.^70^ A similar trend was also reported by Jakkampudi *et al.* in their recent study with SPLUNC1 (short palate lung and nasal epithelial clone 1) AMP derivatives.^93^

### 3.2 Toxicity to eukaryotic cells

The lytic activity of all peptides was systematically examined to determine their potential toxicity to eukaryotic cells, specifically RBCs and WBCs. The obtained data, as summarized in Table 2, shows that neither of the peptides exhibits any discernible toxic effects on eukaryotic cells, highlighting their promising potential for therapeutic applications. This contrasts to helical forming peptides, which usually display some toxicity.^70^

### 3.3 Secondary structure

**Fig. 2:**
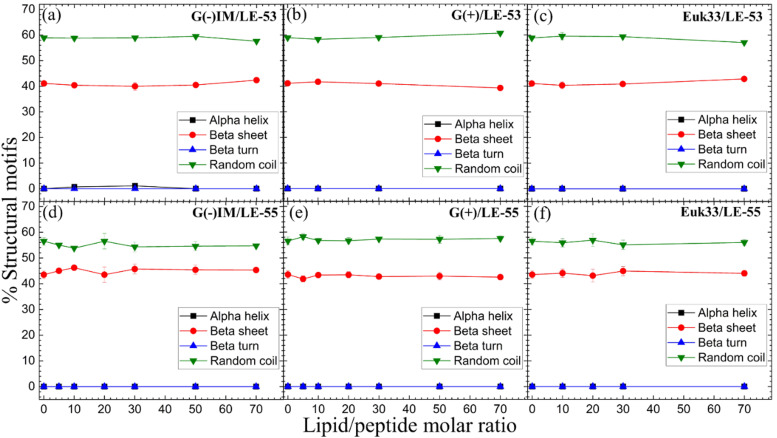
%Structural motifs *vs.* lipid/peptide molar ratio of LE-53 (a)–(c) and LE-55 (d)–(f) in three different LMM ULVs. Standard deviations represent 3–4 fitting results using shape analysis.

CD results of LE-53 and LE-55 in three distinct LMM ULVs are shown in Fig. 2. Fig. S1 (ESI†) presents the MRE data for lipid/peptide molar ratios. A comprehensive summary of the percentage of all four secondary structural motifs observed in the peptides can be found in Tables S1–S6 (ESI†). Results indicate that both LE-53 and LE-55 displayed approximately 55–60% random coil and 40–45% β-sheet structures in their pure form, as well as when interacting with G(−)IM, G(+), and Euk33 LMMs. Despite similar secondary structure, LE-53 exhibited notable bactericidal activity against both G(−) and G(+) bacteria, as demonstrated in Table 2, while LE-55 was less effective.

Antimicrobial peptides are typically short in length, consisting of fewer than 100 amino acids. They possess amphipathic properties due to the presence of cationic and hydrophobic residues. While sharing these common characteristics, AMPs exhibit significant diversity in their primary, secondary, and tertiary structures.^94,95^ The α-helical secondary structure, characterized by hydrophilic residues aligned on one face and hydrophobic residues on the opposite face, facilitates optimal peptide–membrane interactions.^96,97^ For example, peptides derived from Magainin 2, which have higher α-helicity, promote antibacterial activity.^98^ In our recent study α-helicity was found to be LMM dependent, showing a higher helicity in G(−) > G(+) > Euk33 LMMs.^70^ Two peptides, E2-35 and E2-05, rich in R and W residues, exhibited predominantly helical structures in G(−) IM (85–90%) and G(+) LMMs (50–60%), while substitution of R with K (E2-35 → E2-35K) reduced helicity.^70^ The helicity observed in E2-35 and E2-05 showed a strong correlation with their antibacterial efficiency compared to E2-35K.^70^ While α-helicity is often associated with higher efficacy, this is not always the case. For instance, the D8 form of WLBU2, containing 8 valines as the d-enantiomer, displayed a random coil structure in G(−) LMMs, in contrast to WLBU2's predominantly helical structure. Surprisingly, both AMPs exhibited similar efficacy in killing bacteria.^25,99^ Different secondary structures such as β-sheet have also been shown to be efficacious challenging the notion that helicity is crucial for antimicrobial function.^100^

Therefore, based on our results and the broader literature, it appears that the relationship between secondary structure and antibacterial efficacy of AMPs is complex and multifaceted. Factors beyond secondary structure, such as peptides’ physical attributes and location in lipid membranes may play significant roles in determining antimicrobial activity.

### 3.4 Membrane elasticity and lipid chain order parameter

**Fig. 3:**
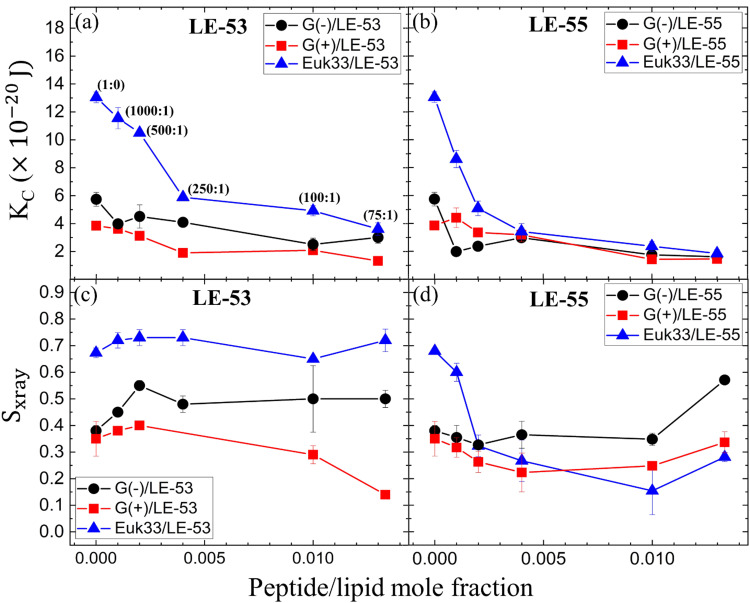
(a) and (b) Bending modulus (*K*_C_) of G(−) IM (black circles), G(+) (red squares) and Euk33 (blue triangles) LMMs interacting with two AMPs as shown. (c) and (d) Chain order parameter (*S*_xray_) of two AMPs with LMMs (colors as in a and b). In (a), lipid-to-peptide molar ratios are denoted within parentheses, and this notation applies to all the subsequent plots (b)–(d). The standard deviations are from duplicate or triplicate samples.

Fig. 3(a) and (b) show the elastic bending modulus parameter (*K*_C_) of G(−) IM (black), G(+) (red) and Euk33 LMMs (blue) in the presence of two AMPs. The *K*_C_ is greatest for Euk33 LMM because it has 33% cholesterol. Research from our lab suggests that cholesterol primarily interacts with the saturated palmitoyl chain in POPC and POPE, which makes these membranes stiffer and organizes the lipid acyl chains.^101–103^ The greater *K*_C_ value for the G(−) control compared to the G(+) control is because of the higher content of PE, as demonstrated by Dupuy *et al.*^66^ A general softening was observed for LE-53 in G(+) and Euk33 LMMs and for LE-55 in all three LMMs, indicating that softening behavior was not significantly different for these peptides and may be unrelated to their different bactericidal efficiency.

What might be more significant is their distinct acyl chain order parameters (*S*_xray_), as illustrated in Fig. 3(c) and (d). Higher *S*_xray_ values indicate well-organized lipid acyl chains, while lower values indicate disorganized chains. The Euk33 control, which contains 33 mol% cholesterol, exhibited the most ordered chains. In contrast, LE-53 initially increased ordering at low concentrations, but at higher concentrations, there was either no change or a decrease in ordering across all three LMMs. This behavior is different from LE-55 which caused initial disordering and some degree of ordering at higher concentraions in all three LMMs. This implies that enhancing the order of lipid chains at a low peptide concentration could play a crucial role in the mechanism of bacterial cell death.

### 3.5 Membrane structural results

**Fig. 4:**
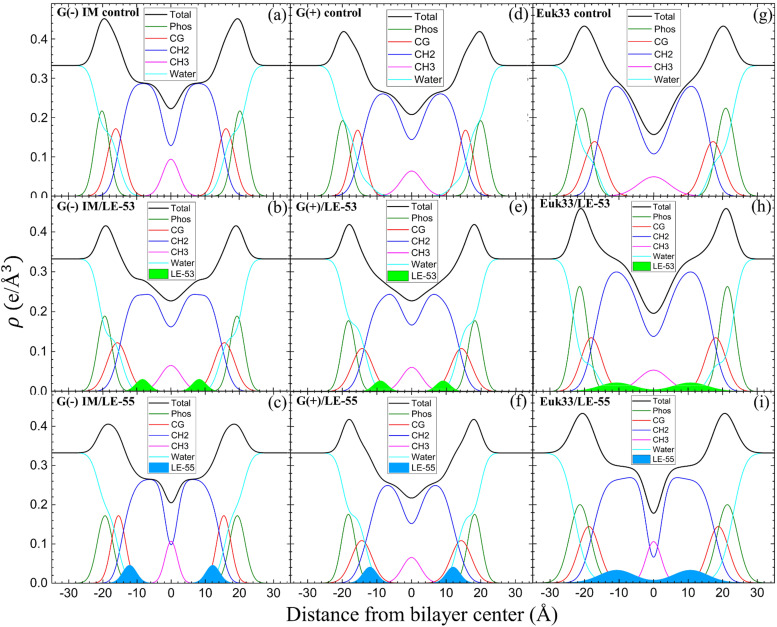
EDPs for G(−) IM LMMs (a)–(c), G(+) LMMs (d)–(f) and Euk33 LMMs (g)–(i) in the presence of LE-53 and LE-55. Component groups in EDPs: phosphate + external headgroup (Phos, green), carbonyl-glycerol (CG, red), CH_2_ (dark blue), CH_3_ (magenta), water (cyan), total (black), LE-53 (filled lime green), LE-55 (filled sky blue). Lipid/peptide molar ratio is 75 : 1.

In this part, we’re investigating the connections between where peptides are located in bilayers and the structural changes of the LMMs in relation to the effectiveness of two peptides in killing bacteria. Fig. S3 (ESI†) shows form factors |*F*(*q*_*z*_)|, while Fig. 4 presents electron density profiles (EDPs) of three LMMs containing a lipid/peptide molar ratio of 75 : 1 for either LE-53 or LE-55. These were determined by fitting XDS form factor data using the scattering density profile (SDP) program.^104^ This program accounts for the volumes of lipids, peptides, and their component groups in the bilayer, along with the number of electrons each component contributes. We fitted the form factors by placing a Gaussian envelope for the peptide in three potential locations: the headgroup, hydrocarbon region, or a combination of both, then assessed the fit quality using chi-square. Generally, the SDP bilayer model fit the XDS form factor data well (Fig. S3, ESI†), resulting in EDPs typical of fully hydrated membranes. The various component groups in EDPs are Phos (phosphate plus outer headgroup), CG (carbonyl/glycerol), CH_2_ (methylene hydrocarbon region containing CH groups), CH_3_ (terminal methyl group), Water (fills volumes around other groups to maintain a total probability of one), and Total (sum of all component groups). Key measures derived from these EDPs include the combined peak-to-peak distance of Phos and CG (*D*_HH_), and the full-width at half-maximal of the hydrocarbon region (2*D*_C_), both of which indicate membrane thickness. The EDP also determines the area per lipid molecule (*A*_L_) using lipid and peptide volumes. A summary of the XDS structural results for the three LMMs used in this study interacting with LE-53 and LE-55 is shown in Table 3.

**Table 3:** Summary of structural results from XDS and the charge/residue

Sample (lipid/peptide (75 : 1))	Area/lipid *A*_L_ [Å^2^] (±1.0)	*D*_HH_ [Å] (±0.5)	2*D*_C_ [Å] (±0.5)	Net charge/residue
G(−) IM/control	70.8	39.2	29.1	—
G(−) IM/LE-53	73.0	38.1	28.9	−0.24
G(−) IM/LE-55	74.6	36.9	28.6	−0.18
G(+)/control	73.4	38.5	28.9	—
G(+)/LE-53	82.5	36.1	26.3	−0.28
G(+)/LE-55	83.5	36.1	26.3	−0.21
Euk33/control	64.0	40.3	32.0	—
Euk33/LE-53	63.1	42.2	33.8	+0.01
Euk33/LE-55	61.8	41.2	35.1	+0.01

**Fig. 5:**
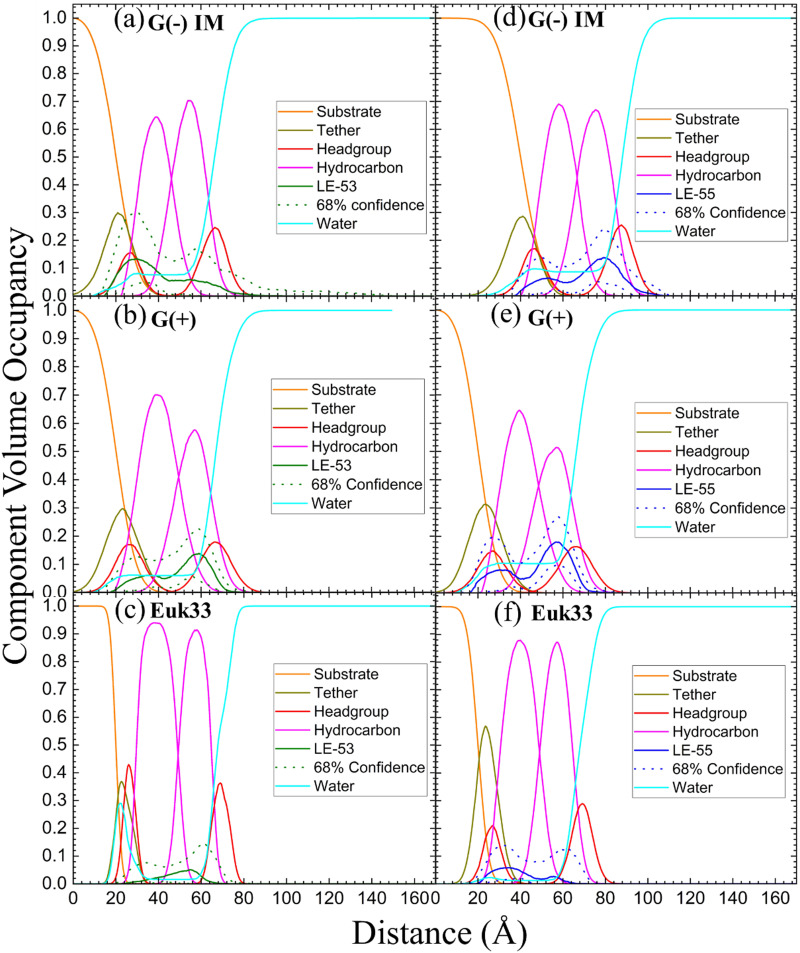
Neutron reflectivity component volume occupancy of LE-53 (a)–(c) and LE-55 (d)–(f) in a single tethered bilayer of G(−) IM, G(+) and Euk33. Component volumes: gold-covered silicon substrate (orange), tether (olive), headgroups (red), hydrocarbons (magenta), LE-53 (green), LE-55 (blue), water (cyan). The dotted lines represent the 68% confidence limit of the composition-space fit.

XDS data reveal that LE-53 locates in the hydrocarbon region, close to the CG headgroup Gaussian in both G(−) and G(+) LMMs (Fig. 4(b) and (e)). LE-55 also locates in the hydrocarbon region of both bacterial LMMs, but closer to the CG headgroup than LE-53 (Fig. 4(c) and (f)). Therefore, LE-53's upper hydrocarbon location correlates with efficient bacterial destabilization. The cause of shallower insertion of LE-55 (8 Rs) in the hydrocarbon region could be its lower hydrophobicity. In addition LE-55 has higher R content compared to LE-53 (6 Rs). The amino acid R contains two extra nitrogens, which allows the guanidinium part of the molecule to form up to six hydrogen bonds.^105^ This unique feature of R enables it to interact with phosphate groups in various ways, forming complexes. A simulation study conducted by Allolio *et al.* discovered that the charged side chains of nona-arginine (R_9_) tend to attach to lipid headgroups, particularly those with negatively charged phosphates.^106^ In our recent study we observed similar behavior with R-rich peptides E2-35 and E2-05.^70^ Therefore, it's more probable for the R-rich peptide LE-55 to be attached to the phosphate groups and negatively charged headgroups of lipids (such as POPG and TOCL) in both Gram-negative and Gram-positive lipid multilayer membranes. The smaller size and higher hydrophobicity of LE-53 (Table 1) might explain its tendency to reside in the upper hydrocarbon region. In order to verify the locations of the peptides, we conducted NR on LE-53 and LE-55. The LMMs were attached to the gold-coated silicon substrate by a lipid tether. NR curves are shown in Fig. S4 (ESI†). Fig. 5 provides a graphical summary of the membrane location of both LE-53 and LE-55 in all three LMMs from NR measurements. These NR results are quantified in Table S7 (ESI†). As illustrated in Fig. 5, the peptides’ locations found by NR are in agreement with the locations determined using XDS.

As indicated in Table 3, both peptides decrease the thickness of the membrane (measured by 2*D*_C_ and *D*_HH_) in both G(−) and G(+) membranes, regardless of their position within the bilayer. Likewise, the peptides increase the area per lipid (*A*_L_) in both types of membranes. Thus, this suggests that changes in membrane thickness and area per lipid are not related to the effectiveness of killing bacteria. Interestingly, LE-53 and LE-55 peptides do not exhibit toxicity towards eukaryotic cells where both peptides localize in the hydrocarbon region of eukaryotic LMMs (Euk33). In our recent studies involving helical peptides rich in Arg and Trp we have observed that peptides which exhibit a certain level of toxicity tend to localize in the headgroup region of the Euk33 bilayer.^70^ Conversely, the non-toxic peptide E2-35K prefers to locate within the hydrocarbon region of the lipid bilayer.^70^ This suggests a correlation between the peptides’ lack of toxicity and their location within the hydrocarbon region of the bilayer.

### 3.6 Fusogenicity of LE peptides

While numerous reports have discussed lipid vesicle fusion induced by peptides,^107–112^ only a limited number of studies have investigated the fusogenicity of AMPs.^113–115^ In this work, we examine the correlation between antibacterial activity and fusogenicity of LE peptides by conducting solution SAXS measurements on G(−)IM, G(+), and Euk33 ULVs in the presence of these peptides.

**Fig. 6:**
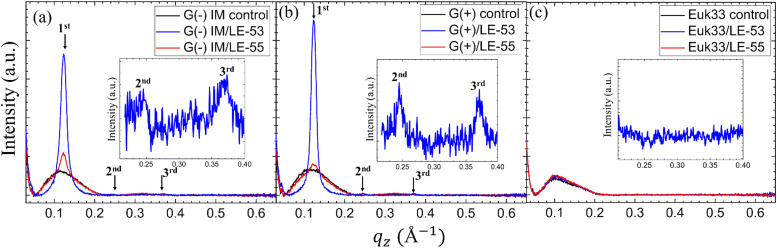
Small angle X-ray scattering profiles of (a) G(−)IM, (b) G(+) and (c) Euk33 ULVs (black) in the presence of LE-53 (blue) and LE-55 (red) AMPs. The arrows indicate Bragg orders. Lipid/peptide molar ratio is 75 : 1. Additionally, (a) and (b) contain insets highlighting the 2nd and 3rd Bragg orders observed in G(−) and G(+) ULVs, and (c) contains inset highlighting no Bragg orders in Euk33 ULVs when incorporated with LE-53.

**Fig. 7:**
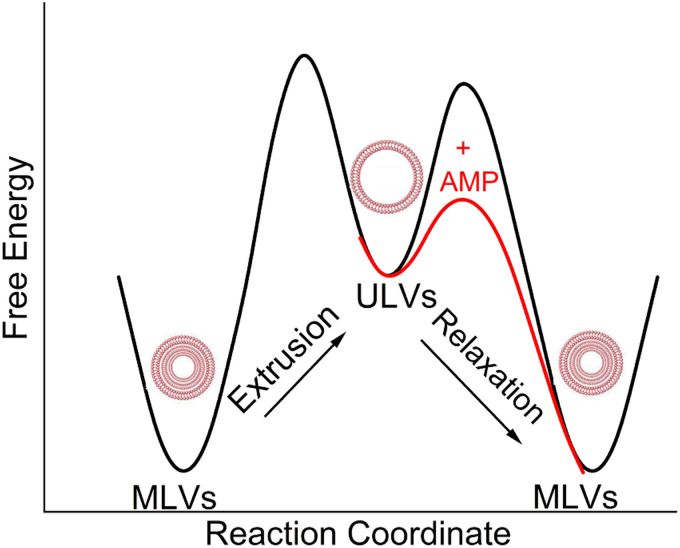
A schematic diagram of free energy *vs.* reaction coordinate, which illustrates the transition of MLVs from a low energy configuration to ULVs in a high energy configuration through extrusion. The diagram also depicts the subsequent transition of ULVs back to MLVs with the addition of AMPs.

The SAXS data (Fig. 6(a)–(c)) obtained from control samples (G(−)IM, G(+), and Euk33) LMM ULVs showed a diffuse modulation of scattered intensity, which is indicative of positionally uncorrelated bilayers commonly found in ULV dispersions. In the presence of LE-53, the ULVs underwent a significant structural transition and exhibited characteristic Bragg, lamellar orders at *q* values of 0.123 Å^−1^, 0.246 Å^−1^ and 0.369 Å^−1^ with D spacing of 51.1 Å for both G(−) and G(+). With the addition of LE-55, only small Bragg orders became visible in the first lobe. This pattern suggests the formation of multilamellar vesicles (MLVs) with lamellar structure. Using the Scherrer equation,^116,117^ LE-53 produces lamellarity of 86 (G(−)) and 107 (G(+)) bilayers while LE-55 produces 30 (G(−)) and 20 (G(+)) bilayers. The transition from ULV to MLV implies vesicle fusion. Free energy changes associated with this process are illustrated in Fig. 7.

Membrane fusion plays a crucial role in various biological processes such as the entry of enveloped viruses into host cells, fertilization, cell division, and synaptic transmission.^113^ This process is facilitated by fusogenic peptide sequences, typically found within larger proteins.^113,118^ Additionally, certain membrane-active peptides, including cell-penetrating peptides (CPPs), possess the capability to induce fusion of membranes and vesicles.^113,119,120^ In interactions between CPPs and complex lipid mixtures such as phosphatidylcholine/PE/bis(monoacylglycerol), not only fusion and leakage, but also leaky fusion (simultaneous fusion and leakage) have been observed.^111,121^ Recently, Allolio *et al.* have shown that arginine-rich CPPs enter vesicles by inducing membrane multilamellarity and fusion.^106^ A combination of magainin 2 and PGLa leads to morphological changes in the membrane that resemble fusion, which could potentially account for their ability to cause leakage.^96^ Leakage activity through leaky vesicle fusion has also been reported as a likely mechanism of membrane permeabilization by an antimicrobial polycation (poly-NM) according to Shi *et al.*^122^ For fusion between vesicles to happen, two obstacles must be surmounted. Firstly, both membranes must be brought into close proximity,^123,124^ a task aided by the neutralization of negative charges on lipid headgroups through the interaction with positively charged peptides.^123,124^ Secondly, the creation of a fusion stalk demands high curvatures of the lipid layers. Various lipid types exhibit different tendencies for forming fusion stalks.^125^ The inherent negative curvatures of POPE^126,127^ and TOCL,^128^ coupled with their well-documented inclination to form non-lamellar structures, contribute to the process of membrane fusion.^125,126,128–131^ This lipid-intrinsic tendency can be further enhanced by the binding and insertion of amphiphiles, such as AMPs.^114,132^ In the present study, it is noteworthy that membrane fusion is more pronounced in the presence of the shorter LE-53 peptide, which exhibits higher efficiency as an antibacterial agent compared to LE-55. This finding raises the possibility of vesicle fusion playing a role in the antimicrobial activity of the peptides. It suggests that induction of curvature may help to disrupt the bacterial membrane. In contrast, it is noteworthy that both LE-55 and LE-53, which are non-toxic to eukaryotic cells, did not induce fusion in the eukaryotic ULVs, as exemplified in Fig. 6(c).

Experiments utilizing X-ray diffuse scattering on lipid bilayer stacks have shed light on the fusion mediation mechanism by observing a significant reduction in membrane bending rigidity upon the addition of small molar fractions of fusion peptides such as FP23,^133^ which localizes in the upper hydrocarbon region.^134^ In addition, studies have highlighted the hydrophobic nature of most fusion peptides, which has been proposed to contribute to the fusion process.^135–137^ Remarkably, the behavior of LE-53, being more hydrophobic than LE-55 and localizing in the upper hydrocarbon region, is similar to these findings.

## 4 Conclusions

This research demonstrates a systematic examination of a possible response to the growing threat of antibiotic resistance. By exploring the potential of AMPs as effective alternatives, the study highlights LE-53 (12 AAs) as a potent solution against both G(−) and G(+) bacteria, all the while ensuring the safety of human cells. The efficacy of LE-53 can be attributed to its elevated hydrophobicity, resulting in notably lower MICs compared to its counterpart, LE-55 (16 AAs), suggesting that physical attributes drive enhanced efficacy. Increased order of lipid acyl chains surfaces as a second determinant for bacterial destabilization. Thirdly, the study unveils the pivotal connection between LE-53's upper hydrocarbon location and its efficacy, shedding light on its mode of action. Fourthly, the most remarkable revelation lies in LE-53's distinctive capacity to trigger vesicle fusion in bacterial model membranes, while remaining inert in eukaryotic LMMs, underscoring a targeted disruption strategy. CD study reveals that LE-53 and LE-55 both maintain random coil and β-sheet structures when in contact with G(−) and G(+) LMMs, which suggests that secondary structure does not distinguish these two AMPs. These exceptional characteristics of LE-53 hold significant promise in addressing antibiotic-resistant strains without compromising human cell integrity.

## Author contributions

Investigation (SM, BC, YL, MC, MM, BE, RK, SH, BK, FH, STN), data curation and formal analysis (SM, BC, YL, MC, MM, BE, RK, FH, STN), conceptualization (SM, STN), resources (BD,STN), funding acquisition (BD, STN), supervision (SM, STN), methodology (FH, STN), Project administration (BD, STN), writing – original draft (SM, STN), writing – review & editing (BK, SH, FH, BD, RK, MC, BE, YL, SM, STN).

## Conflicts of interest

The authors declare no conflict of interest in this work.

## Supplementary Material

SM-020-D4SM00220B-s001

## References

[cit1] Wilson B. A. and Ho B. T., Revenge of the Microbes: How Bacterial Resistance is Undermining the Antibiotic Miracle, John Wiley & Sons, 111 River St, Hoboken, New Jersey, 07030-5773, USA, 2023

[cit2] Årdal C., Balasegaram M., Laxminarayan R., McAdams D., Outterson K., Rex J. H., Sumpradit N. (2020). Nat. Rev. Microbiol..

[cit3] Rizk N. A., Moghnieh R., Haddad N., Rebeiz M.-C., Zeenny R. M., Hindy J.-R., Orlando G., Kanj S. S. (2021). Antibiotics.

[cit4] Murray C. J., Ikuta K. S., Sharara F., Swetschinski L., Aguilar G. R., Gray A., Han C., Bisignano C., Rao P., Wool E. (2022). Lancet.

[cit5] Magana M., Pushpanathan M., Santos A. L., Leanse L., Fernandez M., Ioannidis A., Giulianotti M. A., Apidianakis Y., Bradfute S., Ferguson A. L. (2020). Lancet Infect. Dis..

[cit6] Mookherjee N., Hancock R. (2007). Cell. Mol. Life Sci..

[cit7] Liu Y., Breukink E. (2016). Antibiotics.

[cit8] Sarkar P., Yarlagadda V., Ghosh C., Haldar J. (2017). MedChemComm.

[cit9] Sibinelli-Sousa S., Hespanhol J. T., Bayer-Santos E. (2021). J. Bacteriol..

[cit10] Santos J. A., Lamers M. H. (2020). Antibiotics.

[cit11] Kapoor G., Saigal S., Elongavan. A. (2017). J. Anaesthesiol., Clin. Pharmacol..

[cit12] Fernandez-Villa D., Aguilar M. R., Rojo L. (2019). Int. J. Mol. Sci..

[cit13] Deslouches B., Steckbeck J. D., Craigo J. K., Doi Y., Burns J. L., Montelaro R. C. (2015). Antimicrob. Agents Chemother..

[cit14] Zhu Y., Hao W., Wang X., Ouyang J., Deng X., Yu H., Wang Y. (2022). Med. Res. Rev..

[cit15] Matsuzaki K. (2019). Antimicrob. Pept..

[cit16] Ittig S., Lindner B., Stenta M., Manfredi P., Zdorovenko E., Knirel Y. A., Dal Peraro M., Cornelis G. R., Zähringer U. (2012). PLoS Pathog..

[cit17] Silipo A., Sturiale L., Garozzo D., Erbs G., Jensen T. T., Lanzetta R., Dow J. M., Parrilli M., Newman M. A., Molinaro A. (2008). ChemBioChem.

[cit18] Pelletier M. R., Casella L. G., Jones J. W., Adams M. D., Zurawski D. V., Hazlett K. R., Doi Y., Ernst R. K. (2013). Antimicrob. Agents Chemother..

[cit19] Yang L., Harroun T. A., Weiss T. M., Ding L., Huang H. W. (2001). Biophys. J..

[cit20] Velasco-Bolom J. L., Garduno-Juarez R. (2022). J. Biomol. Struct. Dyn..

[cit21] Uematsu N., Matsuzaki K. (2000). Biophys. J..

[cit22] Woolley G. A., Wallace B. A. (1992). J. Membr. Biol..

[cit23] Hallock K. J., Lee D. K., Ramamoorthy A. (2003). Biophys. J..

[cit24] Rathinakumar R., Wimley W. C. (2008). J. Am. Chem. Soc..

[cit25] Heinrich F., Salyapongse A., Kumagai A., Dupuy F. G., Shukla K., Penk A., Huster D., Ernst R. K., Pavlova A., Gumbart J. C. (2020). Chem. – Eur. J..

[cit26] Chen F. Y., Lee M. T., Huang H. W. (2003). Biophys. J..

[cit27] Aisenbrey C. , Marquette A. and Bechinger B., in Antimicrobial Peptides Basics for Clinical Application, Advances in Experimental Medicine and Biology, ed. K. Matsuzaki, Springer Nature, Singapore, Singapore, 2019, vol. 1117, ch. 4, pp.33–64 10.1007/978-981-13-3588-4_4 30980352

[cit28] Epand R. M., Epand R. F. (2009). Biochim. Biophys. Acta, Biomembr..

[cit29] Shai Y., Oren Z. (2001). Peptides.

[cit30] Chen Y. X., Guarnieri M. T., Vasil A. I., Vasil M. L., Mant C. T., Hodges R. S. (2007). Antimicrob. Agents Chemother..

[cit31] Kang H.-K., Kim C., Seo C. H., Park Y. (2017). J. Microbiol..

[cit32] Zasloff M. (2002). Nature.

[cit33] Park C. B., Yi K.-S., Matsuzaki K., Kim M. S., Kim S. C. (2000). Proc. Natl. Acad. Sci. U. S. A..

[cit34] Park C. B., Kim M. S., Kim S. C. (1996). Biochem. Biophys. Res. Commun..

[cit35] Park C. B., Kim H. S., Kim S. C. (1998). Biochem. Biophys. Res. Commun..

[cit36] Patrzykat A., Friedrich C. L., Zhang L., Mendoza V., Hancock R. E. (2002). Antimicrob. Agents Chemother..

[cit37] Boman H. G., Agerberth B., Boman A. (1993). Infect. Immun..

[cit38] Roy R., Tiwari M., Donelli G., Tiwari V. (2018). Virulence.

[cit39] Lehrer R., Barton A., Daher K. A., Harwig S., Ganz T., Selsted M. E. (1989). J. Clin. Invest..

[cit40] Andreu D., Rivas L. (1998). Pept. Sci..

[cit41] Zhang L.-j, Gallo R. L. (2016). Curr. Biol..

[cit42] Mutschler A., Tallet L., Rabineau M., Dollinger C., Metz-Boutigue M.-H., Schneider F., Senger B., Vrana N. E., Schaaf P., Lavalle P. (2016). Chem. Mater..

[cit43] Mutschler A., Betscha C., Ball V., Senger B., Vrana N. E., Boulmedais F., Schroder A., Schaaf P., Lavalle P. (2017). Chem. Mater..

[cit44] Vidal L., Thuault V., Mangas A., Coveñas R., Thienpont A., Geffard M. (2014). J. Amino Acids.

[cit45] Nishikawa M., Ogawa K. i (2004). Antimicrob. Agents Chemother..

[cit46] Eloïse L., Petit L., Nominé Y., Heurtault B., Kaddour I. B. H., Senger B., Fores J. R., Vrana N. E., Barbault F., Lavalle P. (2024). Eur. J. Med. Chem..

[cit47] Shah S. S., Casanova N., Antuono G., Sabatino D. (2020). Front. Chem..

[cit48] Mitchell D. J., Steinman L., Kim D., Fathman C., Rothbard J. (2000). J. Pept. Res..

[cit49] Aróstica M., Rojas R., Aguilar L. F., Carvajal-Rondanelli P., Albericio F., Guzmán F., Cárdenas C. (2022). Membranes.

[cit50] Derakhshankhah H., Jafari S. (2018). Biomed. Pharmacother..

[cit51] Schmidt N., Mishra A., Lai G. H., Wong G. C. (2010). FEBS Lett..

[cit52] Ruseska I., Zimmer A. (2020). Beilstein J. Nanotechnol..

[cit53] Madani F., Lindberg S., Langel Ü., Futaki S., Gräslund A. (2011). J. Biophys..

[cit54] Tan P., Fu H., Ma X. (2021). Nano Today.

[cit55] Deslouches B., Phadke S. M., Lazarevic V., Cascio M., Islam K., Montelaro R. C., Mietzner T. A. (2005). Antimicrob. Agents Chemother..

[cit56] Stromstedt A. A., Ringstad L., Schmidtchen A., Malmsten M. (2010). Curr. Opin. Colloid Interface Sci..

[cit57] Vogel H. J., Schibli D. J., Jing W., Lohmeier-Vogel E. M., Epand R. F., Epand R. M. (2002). Biochem. Cell Biol..

[cit58] Skinner M., Kiselev A., Isaacs C., Mietzner T., Montelaro R., Lampe M. (2010). Antimicrob. Agents Chemother..

[cit59] Mandell J. B., Koch J. A., Deslouches B., Urish K. L. (2020). J. Orthop. Res..

[cit60] Wimley W. C., White S. H. (1996). Nat. Struct. Biol..

[cit61] Xiang W., Clemenza P., Klousnitzer J., Chen J., Qin W., Tristram-Nagle S., Doi Y., Di Y. P., Deslouches B. (2022). Front. Microbiol..

[cit62] Booth V., Warschawski D. E., Santisteban N. P., Laadhari M., Marcotte I. (2017). Biochim. Biophys. Acta, Proteins Proteomics.

[cit63] Marquette A., Bechinger B. (2018). Biomolecules.

[cit64] Leber R., Pachler M., Kabelka I., Svoboda I., Enkoller D., Vacha R., Lohner K., Pabst G. (2018). Biophys. J..

[cit65] Buck A. K., Elmore D. E., Darling L. E. O. (2019). Future Med. Chem..

[cit66] Dupuy F. G., Pagano I., Andenoro K., Peralta M. F., Elhady Y., Heinrich F., Tristram-Nagle S. (2018). Biophys. J..

[cit67] Kumagai A., Dupuy F. G., Arsov Z., Elhady Y., Moody D., Ernst R. K., Deslouches B., Montelaro R. C., Di Y. P., Tristram-Nagle S. (2019). Soft Matter.

[cit68] Pandit G., Chowdhury N., Mohid S. A., Bidkar A. P., Bhunia A., Chatterjee S. (2021). ChemMedChem.

[cit69] Deslouches B., Gonzalez I. A., DeAlmeida D., Islam K., Steele C., Montelaro R. C., Mietzner T. A. (2007). J. Antimicrob. Chemother..

[cit70] Mitra S., Coopershlyak M., Li Y., Chandersekhar B., Koenig R., Chen M.-T., Evans B., Heinrich F., Deslouches B., Tristram-Nagle S. (2023). Adv. NanoBiomed Res..

[cit71] Wilkinson S. G. , Microbial Lipids, Academic Press, San Diego, CA, 1988

[cit72] Gottfried E. L. (1967). J. Lipid Res..

[cit73] Branzoi I., Iordoc M., Branzoi F., Vasilescu-Mirea R., Sbarcea G. (2010). Surf. Interface Anal..

[cit74] Deslouches B., Steckbeck J. D., Craigo J. K., Doi Y., Mietzner T. A., Montelaro R. C. (2013). Antimicrob. Agents Chemother..

[cit75] Deslouches B., Hasek M. L., Craigo J. K., Steckbeck J. D., Montelaro R. C. (2016). J. Med. Microbiol..

[cit76] Brahms S., Brahms J. (1980). J. Mol. Biol..

[cit77] Tristram-Nagle S. A. (2007). Methods Mol. Biol..

[cit78] Kučerka N., Liu Y., Chu N., Petrache H. I., Tristram-Nagle S., Nagle J. F. (2005). Biophys. J..

[cit79] Lyatskaya Y., Liu Y., Tristram-Nagle S., Katsaras J., Nagle J. F. (2000). Phys. Rev. E: Stat., Nonlinear, Soft Matter Phys..

[cit80] De Gennes P.-G. and Prost J., The physics of liquid crystals, Oxford university press, 1993

[cit81] Mills T. T., Toombes G. E., Tristram-Nagle S., Smilgies D.-M., Feigenson G. W., Nagle J. F. (2008). Biophys. J..

[cit82] M. Inc, 2022

[cit83] Kučerka N., Nieh M.-P., Katsaras J. (2011). Biochim. Biophys. Acta, Biomembr..

[cit84] Dalgliesh R., Langridge S., Plomp J., De Haan V., Van Well A. (2011). Phys. B.

[cit85] Budvytyte R., Valincius G., Niaura G., Voiciuk V., Mickevicius M., Chapman H., Goh H.-Z., Shekhar P., Heinrich F., Shenoy S. (2013). Langmuir.

[cit86] Eells R. , Hoogerheide D. P., Kienzle P. A., Lösche M., Majkrzak C. F. and Heinrich F., in Characterization of Biological Membranes: Structure and Dynamics, ed. M.-P. Nieh, F. A. Heberle and J. Katsaras, 2019, pp.87–130

[cit87] Shekhar P., Nanda H., Lösche M., Heinrich F. (2011). J. Appl. Phys..

[cit88] Heinrich F., Lösche M. (2014). Biochim. Biophys. Acta, Biomembr..

[cit89] Fauchere J. L., Pliska V. (1983). Eur. J. Med. Chem..

[cit90] Eisenberg D., Weiss R. M., Terwilliger T. C. (1982). Nature.

[cit91] Rosenfeld Y., Lev N., Shai Y. (2010). Biochemistry.

[cit92] Stark M., Liu L. P., Deber C. M. (2002). Antimicrob. Agents Chemother..

[cit93] Jakkampudi T., Lin Q., Mitra S., Vijai A., Qin W., Kang A., Chen J., Ryan E., Wang R., Gong Y. (2023). Biomacromolecules.

[cit94] Koehbach J., Craik D. J. (2019). Trends Pharmacol. Sci..

[cit95] Mai X. T., Huang J. F., Tan J. J., Huang Y. B., Chen Y. X. (2015). J. Pept. Sci..

[cit96] Kabelka I., Vacha R. (2021). Acc. Chem. Res..

[cit97] Mojsoska B., Jenssen H. (2015). Pharmaceuticals.

[cit98] Xu X., Lai R. (2015). Chem. Rev..

[cit99] Di Y., Lin Q., Chen C., Montelaro R., Doi Y., Deslouches B. (2020). Sci. Adv..

[cit100] Powers J.-P. S., Hancock R. E. (2003). Peptides.

[cit101] Pan J., Mills T. T., Tristram-Nagle S., Nagle J. F. (2008). Phys. Rev. Lett..

[cit102] Pan J., Tristram-Nagle S., Nagle J. F. (2009). Phys. Rev. E: Stat., Nonlinear, Soft Matter Phys..

[cit103] Mills T. T., Tristram-Nagle S., Heberle F. A., Morales N. F., Zhao J., Wu J., Toombes G. E. S., Nagle J. F., Feigenson G. W. (2008). Biophys. J..

[cit104] Kucerka N., Nagle J. F., Sachs J. N., Feller S. E., Pencer J., Jackson A., Katsaras J. (2008). Biophys. J..

[cit105] Hristova K., Wimley W. C. (2011). J. Membr. Biol..

[cit106] Allolio C., Magarkar A., Jurkiewicz P., Baxova K., Javanainen M., Mason P. E., Sachl R., Cebecauer M., Hof M., Horinek D., Heinz V., Rachel R., Ziegler C. M., Schrofel A., Jungwirth P. (2018). Proc. Natl. Acad. Sci. U. S. A..

[cit107] Domingues T. M., Perez K. R., Riske K. A. (2020). Langmuir.

[cit108] Marx L., Semeraro E. F., Mandl J., Kremser J., Frewein M. P., Malanovic N., Lohner K., Pabst G. (2021). Front. Med. Technol..

[cit109] Sun L., Hristova K., Wimley W. C. (2021). Nanoscale.

[cit110] Brock D. J., Kondow-McConaghy H., Allen J., Brkljača Z., Kustigian L., Jiang M., Zhang J., Rye H., Vazdar M., Pellois J.-P. (2020). Cell Chem. Biol..

[cit111] Yang S.-T., Zaitseva E., Chernomordik L. V., Melikov K. (2010). Biophys. J..

[cit112] Shi S., Markl A. M., Lu Z., Liu R., Hoernke M. (2022). Langmuir.

[cit113] Ferreira A. R., Ferreira M., Nunes C., Reis S., Teixeira C., Gomes P., Gameiro P. (2023). Membranes.

[cit114] Beck K., Nandy J., Hoernke M. (2023). Soft Matter.

[cit115] Sun J., Xia Y., Li D., Du Q., Liang D. (2014). Biochim. Biophys. Acta, Biomembr..

[cit116] Scherrer P. (1918). Nachr. Ges. Wiss. Gottingen.

[cit117] Patterson A. (1939). Phys. Rev..

[cit118] Chernomordik L. V., Kozlov M. M. (2008). Nat. Struct. Mol. Biol..

[cit119] Joardar A., Pattnaik G. P., Chakraborty H. (2022). J. Membr. Biol..

[cit120] Wadhwani P., Reichert J., Bürck J., Ulrich A. S. (2012). Eur. Biophys. J..

[cit121] Brock D. J., Kondow-McConaghy H., Allen J., Brkljača Z., Kustigian L., Jiang M., Zhang J., Rye H., Vazdar M., Pellois J.-P. (2020). Cell Chem. Biol..

[cit122] Shi S., Fan H., Hoernke M. (2022). Nanoscale Adv..

[cit123] Leikin S. L., Kozlov M. M., Chernomordik L. V., Markin V. S., Chizmadzhev Y. A. (1987). J. Theor. Biol..

[cit124] Witkowska A., Heinz L. P., Grubmüller H., Jahn R. (2021). Nat. Commun..

[cit125] Poojari C. S., Scherer K. C., Hub J. S. (2021). Nat. Commun..

[cit126] Kollmitzer B., Heftberger P., Rappolt M., Pabst G. (2013). Soft Matter.

[cit127] Meher G., Chakraborty H. (2019). J. Membr. Biol..

[cit128] Chen Y.-F., Tsang K.-Y., Chang W.-F., Fan Z.-A. (2015). Soft Matter.

[cit129] Navas B. P., Lohner K., Deutsch G., Sevcsik E., Riske K., Dimova R., Garidel P., Pabst G. (2005). Biochim. Biophys. Acta, Biomembr..

[cit130] Smirnova Y. G., Risselada H. J., Müller M. (2019). Proc. Natl. Acad. Sci. U. S. A..

[cit131] Koller D., Lohner K. (2014). Biochim. Biophys. Acta, Biomembr..

[cit132] Mishra A., Lai G. H., Schmidt N. W., Sun V. Z., Rodriguez A. R., Tong R., Tang L., Cheng J., Deming T. J., Kamei D. T. (2011). Proc. Natl. Acad. Sci. U. S. A..

[cit133] Tristram-Nagle S., Nagle J. F. (2007). Biophys. J..

[cit134] Tristram-Nagle S., Chan R., Kooijman E., Uppamoochikkal P., Qiang W., Weliky D. P., Nagle J. F. (2010). J. Mol. Biol..

[cit135] Shchelokovskyy P., Tristram-Nagle S., Dimova R. (2011). New J. Phys..

[cit136] Brasseur R., Vandenbranden M., Cornet B., Burny A., Ruysschaert J.-M. (1990). Biochim. Biophys. Acta, Biomembr..

[cit137] Colotto A., Martin I., Ruysschaert J.-M., Sen A., Hui S., Epand R. (1996). Biochemistry.

