# Supplementary material for: Novel non-helical antimicrobial peptides insert into and fuse lipid model membranes
Source: Soft Matter. 2024 Apr 29;20(20):4088–101. doi: 10.1039/d4sm00220b (PMC11109824; doi:10.1039/d4sm00220b)
Supplement: SM-020-D4SM00220B-s001 [file SM-020-D4SM00220B-s001.pdf]

Electronic Supplementary Material (ESI) for Soft Matter.
This journal is © The Royal Society of Chemistry 2024

Supplementary Information

Novel non-helical antimicrobial peptides insert into and fuse lipid model membranes

Saheli Mitra[1], Bhairavi Chandersekhar[1], Yunshu Li[1], Mark Coopershlyak[1], Margot E. Mahoney[1], Brandt Evans[1], Rachel Koenig[1], Stephen C. L. Hall[2], Beate Klösgen[3], Frank Heinrich[1,4], Berthony Deslouches[5], Stephanie Tristram-Nagle[1*]

[1]*Biological Physics Group, Physics Department, Carnegie Mellon University, Pittsburgh, PA 15213, USA;* [2]*ISIS Neutron and Muon Source, Rutherford Appleton Laboratory, Harwell Campus, Didcot, Oxfordshire, OX11 0QX, UK;* [3]*University of Southern Denmark, Dept. Physics, Chemistry & Pharmacy, PhyLife, Campusvej 55, ODENSE M 5230, Denmark;* [4]*Center for Neutron Research, National Institute of Standards and Technology, Gaithersburg, MD 20899, USA;*[5]*Department of Environmental and Occupational Health, University of Pittsburgh, Pittsburgh, PA 15261,USA*

*Corresponding author's email: stn@cmu.edu

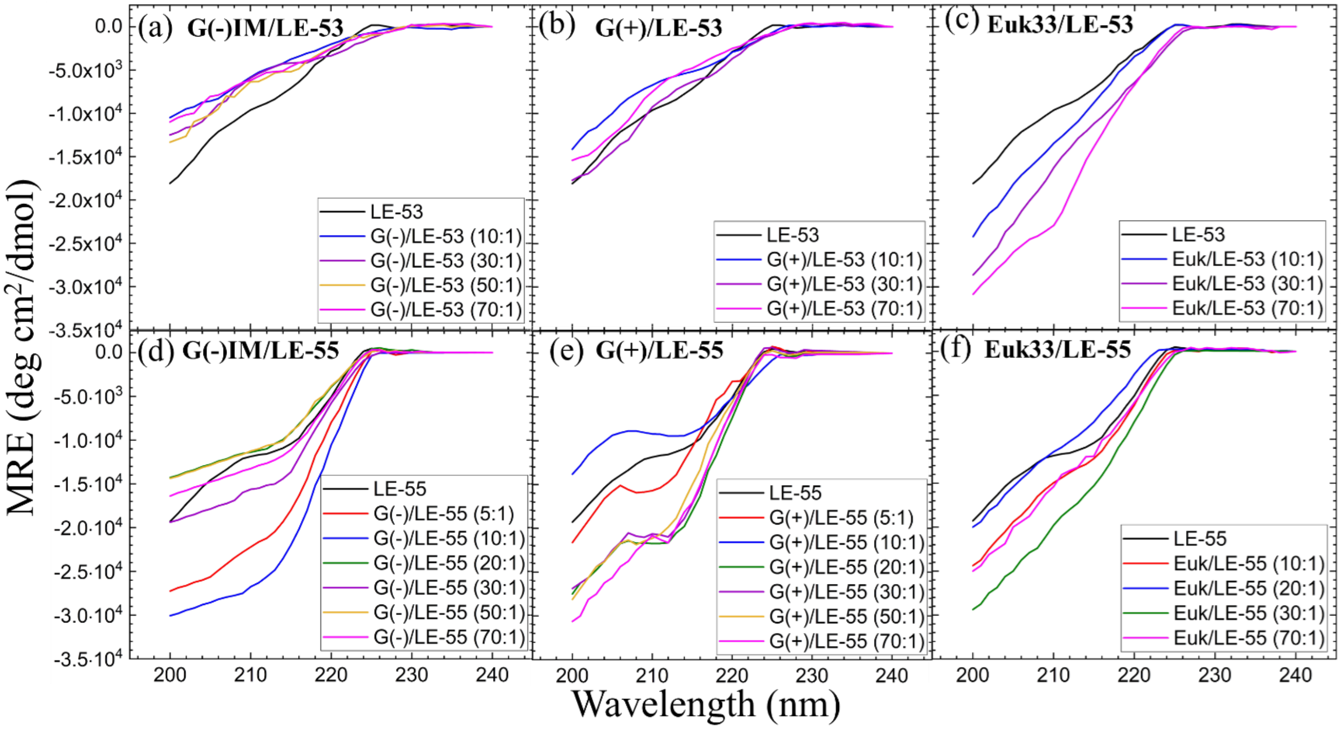

Fig. S1 MRE results of LE-53 (**a, b and c**) and LE-55 (**d, e and f**) in G(-)IM, G(+) and Euk33 LMM ULVs.

Tables S1 – S6 summarize secondary structural results (% α-helix, β-sheet, β-turn and random coil) for LE-53 and LE-55 peptides in three LMMs. R^2 indicates the goodness of fit. Std. devs. were generally ~5-7 % of the values shown.

Table S1. LE-53 CD results of secondary structure in G(-) IM LMMs

G(-) IM/LE-53	α-helix (%)	β-sheet (%)	β-turn (%)	Random	R^2
0:1	0 ± 0	38.9 ± 1.1	0 ± 0	61.1 ± 1.1	0.99
10:1	12.1 ± 2.6	26.2 ± 2.5	0 ± 0	61.4 ± 1.4	0.98
20:1	0 ± 0	41.4 ± 0.8	0 ± 0	58.6 ± 0.8	0.98
30:1	0 ± 0	39.6 ± 0.2	0 ± 0	60.4 ± 0.2	0.98
50:1	0 ± 0	39.2 ± 1.0	0 ± 0	60.8 ± 1.0	0.99

Table S2. LE-53 CD results of secondary structure in G(+) LMMs

G(+)/LE-53	α-helix (%)	β-sheet (%)	β-turn (%)	Random	R^2
0:1	0 ± 0	41.1 ± 0.5	0 ± 0	58.9 ± 0.5	0.99
10:1	0 ± 0	41.7 ± 0.4	0 ± 0	58.3 ± 0.4	0.99
30:1	0 ± 0	41.0 ± 0.4	0 ± 0	59.0 ± 0.4	0.99
70:1	0 ± 0	39.3 ± 0.6	0 ± 0	60.7 ± 0.6	0.99

Table S3. LE-53 CD results of secondary structure in Euk33 LMMs

Euk33/LE-53	α-helix (%)	β-sheet (%)	β-turn (%)	Random	R^2
0:1	0 ± 0	41.1 ± 0.5	0 ± 0	58.9 ± 0.5	0.99
10:1	0 ± 0	40.4 ± 1.4	0 ± 0	59.6 ± 1.4	0.99
30:1	0 ± 0	40.9 ± 0.0	0 ± 0	59.4 ± 0.4	0.99
70:1	0 ± 0	42.9 ± 0.7	0 ± 0	57.1 ± 0.7	0.99

Table S4. LE-55 CD results of secondary structure in G(-) IM LMMs

G(-) IM/LE-55	α-helix (%)	β-sheet (%)	β-turn (%)	Random	R^2
0:1	0 ± 0	43.5 ± 1.5	0 ± 0	56.5 ± 1.5	0.96
5:1	0 ± 0	45.0 ± 0.1	0 ± 0	55.0 ± 0.1	0.97
10:1	0 ± 0	46.2 ± 0.3	0 ± 0	53.8 ± 0.3	0.94
20:1	0 ± 0	43.5 ± 3.0	0 ±0	56.5 ± 3.0	0.98
30:1	0 ± 0	45.7 ± 1.9	0 ± 0	54.3 ± 1.9	0.97
50:1	0 ± 0	45.4 ± 1.8	0 ± 0	54.6 ± 1.8	0.97
70:1	0 ± 0	45.3 ± 0.4	0 ± 0	54.7 ± 0.4	0.96

Table S5. LE-55 CD results of secondary structure in G(+) LMMs

G(+)/LE-55	α-helix (%)	β-sheet (%)	β-turn (%)	Random	R^2
0:1	0 ± 0	43.5 ± 1.5	0 ± 0	56.5 ± 1.5	0.96
5:1	0 ± 0	41.8 ± 1.2	0 ± 0	58.2 ± 1.2	0.98
10:1	0 ± 0	43.3 ± 0.9	0 ± 0	56.7 ± 0.9	0.99
20:1	0 ± 0	43.4 ± 1.2	0 ± 0	56.6 ± 1.2	0.96
30:1	0 ± 0	42.7 ± 0.2	0 ± 0	57.3 ± 0.2	0.98
50:1	0 ± 0	42.9 ± 1.5	0 ± 0	57.2 ± 1.5	0.97
70:1	0 ± 0	42.5 ± 1.0	0 ± 0	57.5 ± 1.0	0.97

Table S6. LE-55 CD results of secondary structure in Euk33 LMMs

Euk33/LE-55	α-helix (%)	β-sheet (%)	β-turn (%)	Random	R^2
0:1	0 ± 0	43.5 ± 1.5	0 ± 0	56.5 ± 1.5	0.96
10:1	0 ± 0	44.1 ± 1.6	0 ± 0	55.9 ± 1.6	0.99
20:1	0 ± 0	43.1 ± 2.5	0 ± 0	56.9 ± 2.5	0.99
30:1	0 ± 0	44.9 ± 1.8	0 ± 0	55.1 ± 1.8	0.99
70:1	0 ± 0	44.0 ± 1.1	0 ± 0	56.0 ± 1.1	0.99

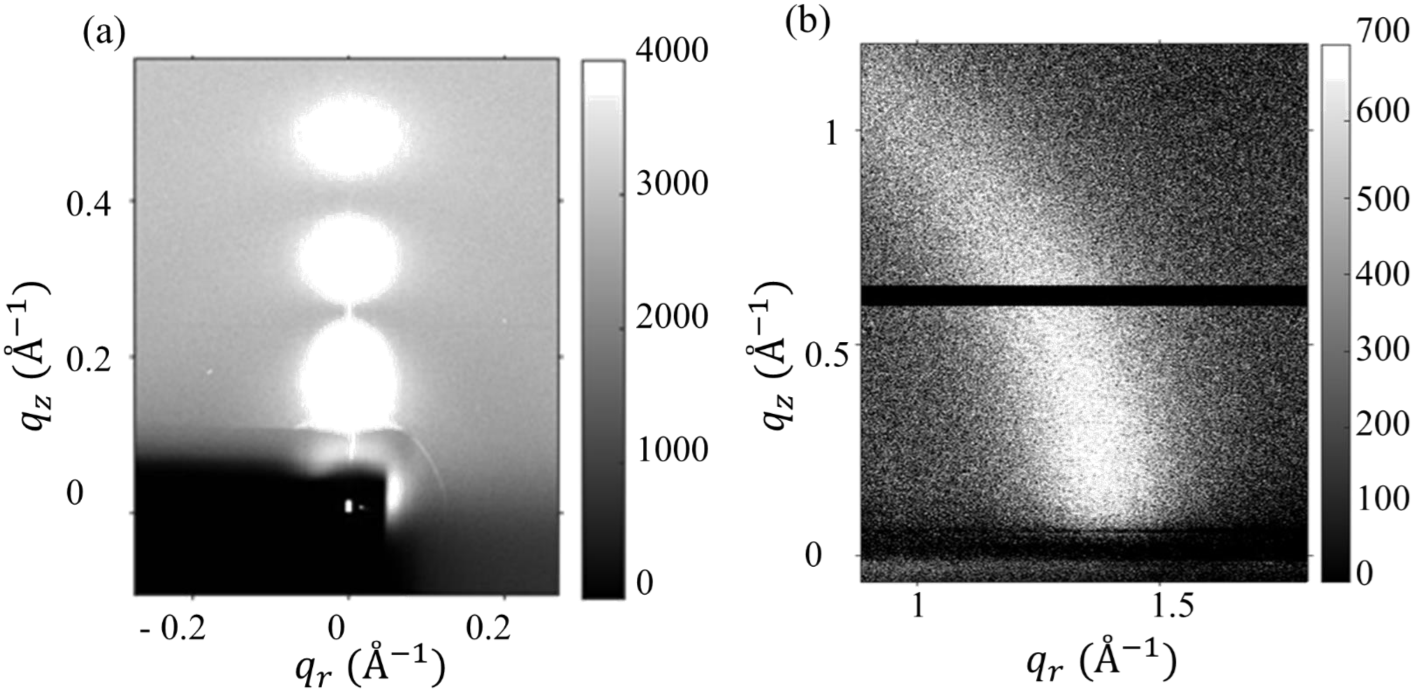

Fig. S2 XDS data obtained at CHESS. Results of 250:1 G(-)IM/LE-53 at 37 ℃. (**a**) Low angle x-ray scattering (LAXS). The sample is fully hydrated with a D-spacing of 102 Å. Three lobes of diffuse x-ray scattering result from fluctuations in the oriented stack of membranes at high hydration. (**b**) Wide angle x-ray scattering (WAXS). The chain correlation is the intensity centered at $q_r \cong 1.4$ Å^{-1} which corresponds to ≅4.5 Å d-spacing. Light grey indicates positive intensity values (see color bars). Black line contains no intensity information and was removed during data analysis.

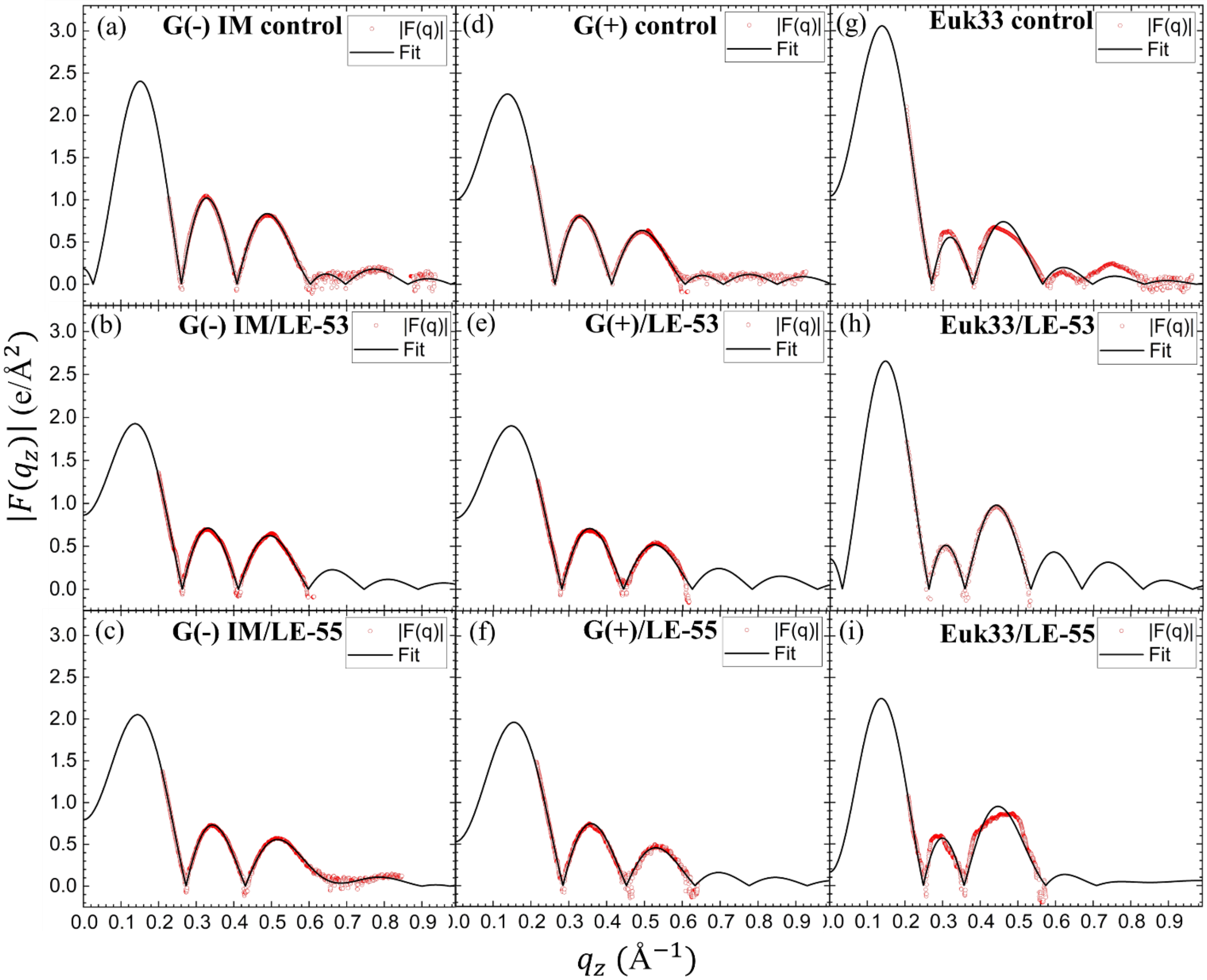

Fig. S3 $|F(q_z)|$ for G(-) IM LMMs (**a – c**), G(+) LMMs (**d - f**) and Euk33 LMMs (**g - i**) in the presence of LE-53 and LE-55. The red points are experimental data, and the black line is the SDP model fit to the data. Lipid/peptide molar ratio is 75:1.

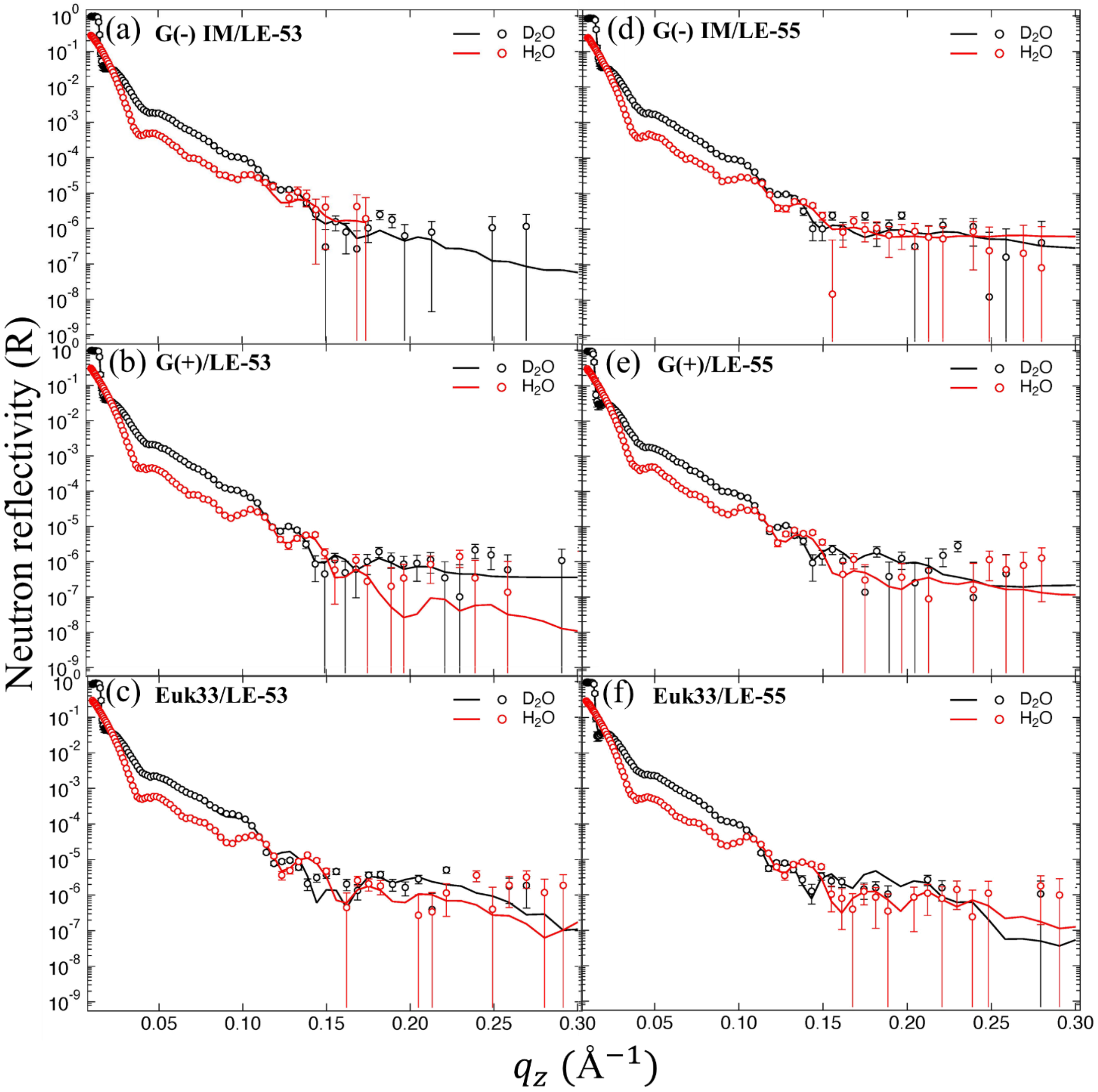

Fig. S4 Neutron reflectivity scattering length profiles of G(-) IM LMMs (**a and d**), G(+) LMMs (**b and e**) and Euk33 LMMs (**c and f**) in the presence of LE-53 and LE-55. The solid black and red lines are the fits to the D_2O and H_2O data respectively.

Table S7. NR results of LE-53 and LE-55 in G(-)IM, G(+) and Euk33 LMMs.

Parameter	G(-)/LE-53	G(+)/LE-53	G(-)/LE-55	G(+) LE-55	Euk33 LE-53	Euk33 LE-55
**Substrate**						
SiOx nSLD / 10^{-6} Å^{-2}	3.2 ± 0.2	3.3 ± 0.2	3.3 ± 0.1	3.3 ± 0.2	3.2 ± 0.1	3.2 ± 0.1
SiOx thickness / Å	24 ± 13	21 ± 12	24 ± 11	25 ± 15	25 ± 14	25 ± 14
Cr nSLD / 10^{-6} Å^{-2}	3.3 ± 0.4	3.1 ± 0.2	3.3 ± 0.3	3.3 ± 0.5	3.3 ± 0.4	3.2 ± 0.4
Cr thickness / Å	26 ± 9	26 ± 11	24 ± 9	24 ± 9	26 ± 12	25 ± 11
Au nSLD / 10^{-6} Å^{-2}	4.5 ± 0.1	4.4 ± 0.1	4.52 ± 0.09	4.67 ± 0.07	4.65 ± 0.09	4.5 ± 0.1
Au thickness / Å	168 ± 6	169 ± 3	169 ± 4	169 ± 5	171 ± 3	168 ± 5
R.M.S. roughness / Å	8 ± 2	8 ± 1	9 ± 2	7 ± 1	2.4 ± 0.3	4 ± 1
**Bilayer**						
R.M.S. roughness / Å	5 ± 1	7 ± 1	6 ± 1	7 ± 2	2.6 ± 0.5	4 ± 1
Tether thickness / Å	10 ± 2	10 ± 2	9 ± 2	10 ± 2	10 ± 1	10 ± 2
Hydrocarbon thickness inner lipid leaflet / Å	16 ± 4	19 ± 3	17 ± 4	18 ± 3	19 ± 3	19 ± 3
Hydrocarbon thickness outer lipid leaflet / Å	16 ± 4	14±3	17 ± 4	14 ± 3	16 ± 4	15 ± 3
Area per lipid, outer leaflet / Å^2	70 ± 20	90±20	70 ± 20	80 ± 20	60 ± 10	60 ± 10
Bilayer completeness / %	90 ± 8	94 ± 5	92 ± 6	92 ± 6	97 ± 3	98 ± 2
**Peptide**						
Amount of membrane-associated protein / Å^3/Å^2	6 ± 3	4 ± 2	5±2	5 ± 2	2 ± 1	3 ± 1
Fraction of protein in hydrocarbons	0.51±0.18	0.70±0.13	0.62±0.12	0.66 ± 0.13	0.80±0.18	0.68±0.16
Fraction of protein in headgroups	0.24±0.08	0.23±0.08	0.23±0.07	0.24 ± 0.06	0.17±0.16	0.24±0.12
Fraction of protein in bulk solvent	0.19±0.14	0.04±0.04	0.10±0.08	0.05 ± 0.04	0.02±0.02	0.02±0.02
Peak position from headgroup / solvent interface / Å	-16±6	-14±4	-13±5	-13 ± 3	-23 ± 11	-16 ± 6
**General**						
Goodness of fit, chi-squared	1.4	0.9	1.3	2.2	4.0	2.4

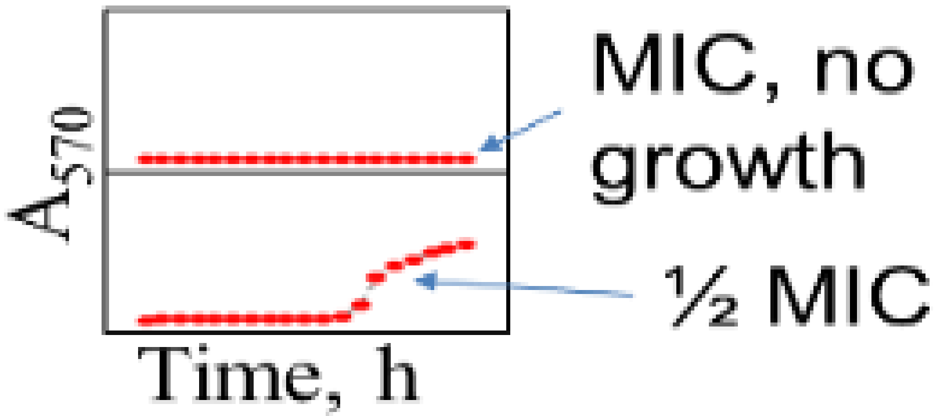

Fig. S5 Assessment of MIC: using a plate reader bacterial turbidity is detected every hour, represented by a red dot, for 18 hours. The growth kinetics, shown as a flat line moved higher, indicate no bacterial growth. The lowest concentration corresponding to a flat growth kinetics line is the MIC or minimum inhibitory concentration.
